# Generalised Analog LSTMs Recurrent Modules for Neural Computing

**DOI:** 10.3389/fncom.2021.705050

**Published:** 2021-09-28

**Authors:** Kazybek Adam, Kamilya Smagulova, Alex James

**Affiliations:** ^1^Department of Electronics and Nanoengineering, Alto University, Espoo, Finland; ^2^Department of Intelligent Systems and Cybersecurity, Astana IT University, Nursultan, Kazakhstan; ^3^School of Electronic Systems and Automation, Digital University Kerala, Trivandrum, India

**Keywords:** analog LSTM, crossbar, memristors, general-purpose LSTM, neural networks

## Abstract

The human brain can be considered as a complex dynamic and recurrent neural network. There are several models for neural networks of the human brain, that cover sensory to cortical information processing. Large majority models include feedback mechanisms that are hard to formalise to realistic applications. Recurrent neural networks and Long short-term memory (LSTM) inspire from the neuronal feedback networks. Long short-term memory (LSTM) prevent vanishing and exploding gradients problems faced by simple recurrent neural networks and has the ability to process order-dependent data. Such recurrent neural units can be replicated in hardware and interfaced with analog sensors for efficient and miniaturised implementation of intelligent processing. Implementation of analog memristive LSTM hardware is an open research problem and can offer the advantages of continuous domain analog computing with relatively low on-chip area compared with a digital-only implementation. Designed for solving time-series prediction problems, overall architectures and circuits were tested with TSMC 0.18 μm CMOS technology and hafnium-oxide (*HfO*_2_) based memristor crossbars. Extensive circuit based SPICE simulations with over 3,500 (inference only) and 300 system-level simulations (training and inference) were performed for benchmarking the system performance of the proposed implementations. The analysis includes Monte Carlo simulations for the variability of memristors' conductance, and crossbar parasitic, where non-idealities of hybrid CMOS-memristor circuits are taken into the account.

## 1. Introduction

The intelligent sensing and information processing at edge is an emerging topic of study in industrial automation. Multiple sensors collect a variety of information in industrial applications, that require sensors to incorporate co-processors for real-time detection, monitoring and data processing. By incorporating neural networks in sensor hardware, it will be possible to embedded intelligent information processing to be low power, high speed, distributed and scalable. Long short-term memory (LSTM) (Hochreiter and Schmidhuber, 1997) that is known to bypass exploding or vanishing gradient problems of Recurrent Neural Network (RNN) find use in a range of time-series prediction and classification problems.

The implementations of LSTM with conventional microprocessors shows that it can take long delays and high power consumption. Some of the existing digital implementations uses FPGAs (Chang et al., 2015; Ferreira and Fonseca, 2016; Guan et al., 2017; Han et al., 2017; Zhang et al., 2017; Chen et al., 2018; Rizakis et al., 2018) and custom built chips (digital ASICs) (Conti et al., 2018; Giraldo and Verhelst, 2018). As can be expected, the digital ASICs are smaller in the area and is more efficient in terms of latency and power consumption than FPGA-implemented hardware. However, even smaller and more efficient chips can be fabricated using memristive crossbar circuits (Li et al., 2019) where weights and vector matrix multiplications (VMM) operations are stored and performed, respectively, using the crossbar arrays.

The full-analog design of the LSTM is an open problem. In this paper, we use memristive crossbars and a set of control circuits for designing, benchmarking and comparing LSTM neural networks. Majority of sensors capture information in analog form. Afterwards the data is digitised and processed by digital processors. As opposed to building LSTM as a co-processing unit away from the sensing unit, we aim to build LSTM for near-sensor processing, suitable for real-time systems. In other words, our system can handle analog signals which allows to avoid analog-to-digital conversion. All the simulations were in SPICE on LSTM architectures for three different time-series prediction problems for performance analysis.

This paper can be read as having a section 2 that provides the architecture and method details, while section 3 highlights the main results. Section 4 provides the summary, while the Supplementary Material provides supporting details and additional results required to reconstruct the results.

## 2. Methodology and Proposed LSTM Design

In this paper, three problems were selected to validate the proposed general purpose LSTM analog information processing architecture elaborated in section 2.2. Problem 1 consists of predicting the number of airline passengers, Problem 2 that of prediction of volcanic *CO*_2_ emission volumes and Problem 3 to predict the Semiconductor Wafer quality. The proposed architecture can be configured to different LSTM architectures required to optimally solve the problems 1–3.

### 2.1. Selected Models and System Level Simulation Setup

The utilised models consists LSTM units followed by a dense layer. Table 1 shows the summary of datasets used in the problem 1, 2, and 3. For problem 1, there are 144 sample points that are converted to 142 datasets. Each of this dataset has one target value and 2 samples points. Problem 2 data was similarly rearranged, while problem 3 was already in the right shape for the selected model. In all the datasets, normalisation is applied to limit the range pf data samples to [0, 1].

**Table 1:** The network configurations used for three problems addressed using the proposed general purpose LSTM hardware.

	**Problem 1**	**Problem 2**	**Problem 3**
Configuration of network [L1(units)+L2(units)]	LSTM(4)+Dense(1)	LSTM(4)+Dense(1)	LSTM(4)+Dense(1)
Train/Test data ratio	2/1	2/1	6.164
Look-back no.	2	2	152
Size of Dataset i.e., (features, samples)	(1, 3= 1*2+1)	(1, 3= 1*2+1)	(1,153= 1*152+1)
Total # of Datasets	142	190	7,164
Epoch size (weight extraction/analysis)	500/300	500/300	180/25
Batch size (weight extraction/analysis)	1/1	1/1	15/1
Weight Constraints	[−1, 1]	[−1, 1]	[−1, 1]
Range of input values	[0, 1]	[0, 1]	[−0.5, 0.5]

Normalisation is performed to adjust to the scale of the input voltage range required for memristor crossbar arrays. The trained weights of LSTM are mapped to memristances of crossbar nodes. The training ratios for each of the problem is shown in Table 1. In the first two problems the train to test ratio of 2/1 is used. As for the third problem, it was chosen according to Wafer (2018) to be 6,164 training datasets over 1,000 testing datasets. Each dataset in problem 3 contains 152 elements (measurement data) and a single label (class). The same model was used for both problem 1 and problem 2. Interestingly, the same network configuration worked well for the third problem despite it using significantly more time-steps to predict the class of a wafer. However, due to large training data availability in problem 3, an epoch size of 180 instead of 500 was used. When analysis is performed on multiple LSTM architectures, the first two problems uses an optimal epoch size of 300, that helps to speed-up Monte Carlo (MC) simulations. The last problem used 180 and 25 epoch sizes for weights extraction and system-level analysis respectively. The numbers here were chosen to be significantly lower due to reduce the training time. The epoch size of 180 was compensated by having batch size of 15.

### 2.2. General Purpose Voltage Driven LSTM Crossbar Architecture

The voltage driven analog LSTM architecture offers high accurate computations with a design of activation function and voltage buffers. In contrast, current based designs of activation function leads to lower computational accuracy.It is mainly due to the cost of designing high accuracy current mirrors aimed to pass both positive and negative currents from one stage to another while isolating the parasitic interaction between the two stages.

**Figure 1:**
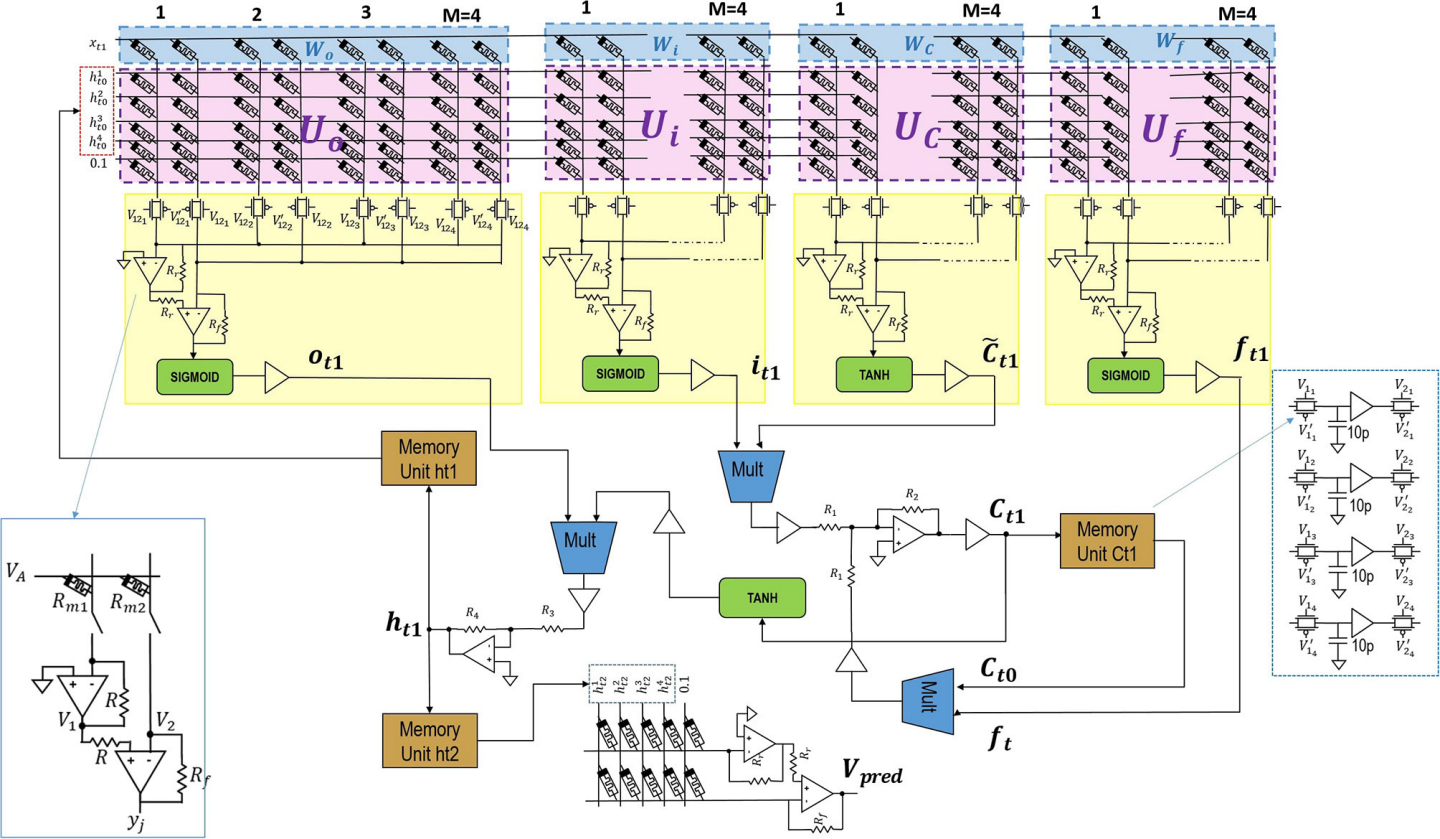
The overall circuit design blocks for the proposed system. The design has No peephole (NP) LSTM with dense layers. The two columns represents a single neuron in the crossbar as seen in left blue box and memory units configuration is shown in right blue box.

The system in the Figure 1 is comprised of LSTM and dense layers^1^. The background equations and notations are included in Supplementary Material for reference. The LSTM layer consists of LSTM unit with M=4 hidden units *h*^1^, *h*^2^, *h*^3^, *h*^4^ and a single unit dense layer without an activation function. Considering bias weights, the size of LSTM weight matrix should be [6 ×16]. Since two memristors per weight are used, the resulting size of the LSTM crossbar is [6 ×32]. Similarly, the size of a crossbar in dense layer is [5,2]. At time *t*_1_ current input of LSTM unit is *x*_*t*1_ is concatenated with ${\vec{h}}_{t0}$ =[0,0,0,0] and enters a crossbar. Crossbar outputs are squashed by hyperbolic tangent or Sigmoid activation circuits and produce *f*_*t*1_, ${\tilde{C}}_{t1}$, *i*_*t*1_, and *o*_*t*1_ values. In turn, they generate output ${\vec{h}}_{t1}$ which is then stored in a *Memory Unit* *h*_*t*1_. Similarly, at time step *t*_2_ the inputs are current input *x*_*t*2_ and the previous unit's output ${\vec{h}}_{t1}$. A new output ${\vec{h}}_{t2}$ is stored in a *Memory Unit* *h*_*t*2_ and passed to the dense layer to produce *x*_*t*3_ which corresponds to *V*_*pred*_. Here, the LSTM layer consists of two cycles of operations before being captured at memory unit *h*_*t*2_ and computed at dense layer.

The positive and negative signs required for *h*_*t*_ is obtained by inversions performed by activation function circuit. The optimised low-power three-stage Class-AB opamps (Saxena and Baker, 2010) and squarer multipliers (Li, 2000) are used. These designs helps to increase the accuracy of implementing LSTM in analog hardware. The resistance ratios for *R*_2_/*R*_1_ and *R*_4_/*R*_3_ are kept at 10, and the crossbar input voltage limited to [−0.1, 0.1].

#### 2.2.1. The Multiply and Accumulate Circuit

The crossbar circuit has voltage as inputs along rows, the nodes having conductance, and output as currents read out along columns. The current here represents a weighted summation operation reflecting a multiply and accumulate operation, or otherwise also known as vector matrix multiplication (VMM). The opamps are used as read out circuit along the columns, and can be used as a current to voltage converter. The use of single opamp with a single column limits the weight mapping to positive values. To take into account negative weights, a single node in crossbar is implemented using conductance of two memristors in two adjacent columns. This translates to two columns per neuron (Hasan et al., 2017). The two opamp design that provides higher robustness is shown in Figure 1. The pass-transistor switches are used to realise VMM sequentially. The *y*_*j*_ are the output nodes, with *R* = 1.25*kΩ* and *Rf* = 1*kΩ*/1.24*kΩ*. The two opamp configuration uses inverting amplifier and summing amplifier to implement a stable difference operation, and voltage amplified with *R*_*f*_ settings.

In addition, it eliminates the usage of extra op-amp inverters that are used for correcting input voltage signs. This is done by swapping the memristance states of memristor pairs in a row. Then the input to the row can be uninverted, if it has an opposite sign. The crossbar design in Figure 1 is implemented in sequential mode, with only the need for two opamps per crossbar. This helps to reduce the area requirement on the chip when implemented.

#### 2.2.2. Activation Function Circuits

**Figure 2:**
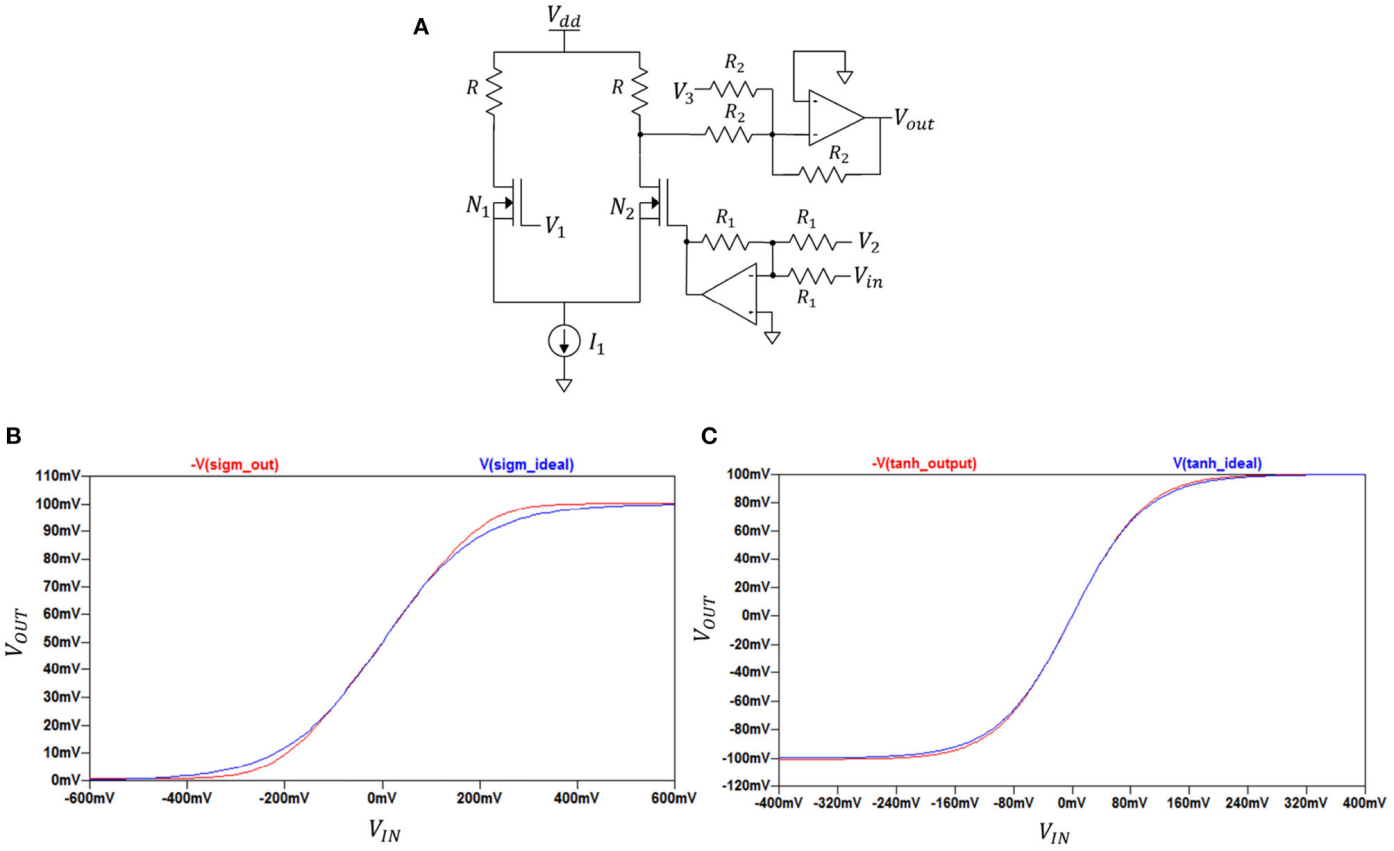
**(A)** Activation function circuit; **(B)** Sigmoid function obtained by circuit from **(A)**; **(C)** Hyperbolic tangent function obtained by circuit from **(A)**.

By adjusting the parameters of the circuit configuration shown in Figure 2A we can realise both sigmoid and hyperbolic tangent functions. The basic idea is to employ the characteristic of differential amplifiers giving sigmoidal output shape in their DC transfer characteristics when sweeping their input voltage difference over a range. The sigmoid or hyperbolic tangent functions are fitted by adjusting the range along the x-axis with help of the bottom-part op-amp and voltage sources *V*_1_ and *V*_2_. The matching along the y-axis is made using the top-part op-amp and voltage source *V*_3_. The form or curvature is set by the following parameters: supply voltage *V*_*dd*_, current *I*_1_, and the sizes of NMOS transistors *N*_1_ and *N*2.

In this figure, *V*_*out*_ is equal to either V(sigm_out) or V(tanh_out) which are output voltages depending on the selected set of parameters. DC transfer characteristics (−V(sigm_out), −V(tanh_out) against *V*_*IN*_) for sigmoid and hyperbolic tangent function circuits along with ideal plots (V(sigm_ideal), V(tanh_ideal) against *V*_*IN*_) for each function are shown in Figures 2B,C. The negative part of each −V(sigm_out) and −V(tanh_out) is canceled at later stages and was kept in mind when designing the overall circuit design.

#### 2.2.3. Four-Quadrant Analog Multiplier Circuit

**Figure 3:**
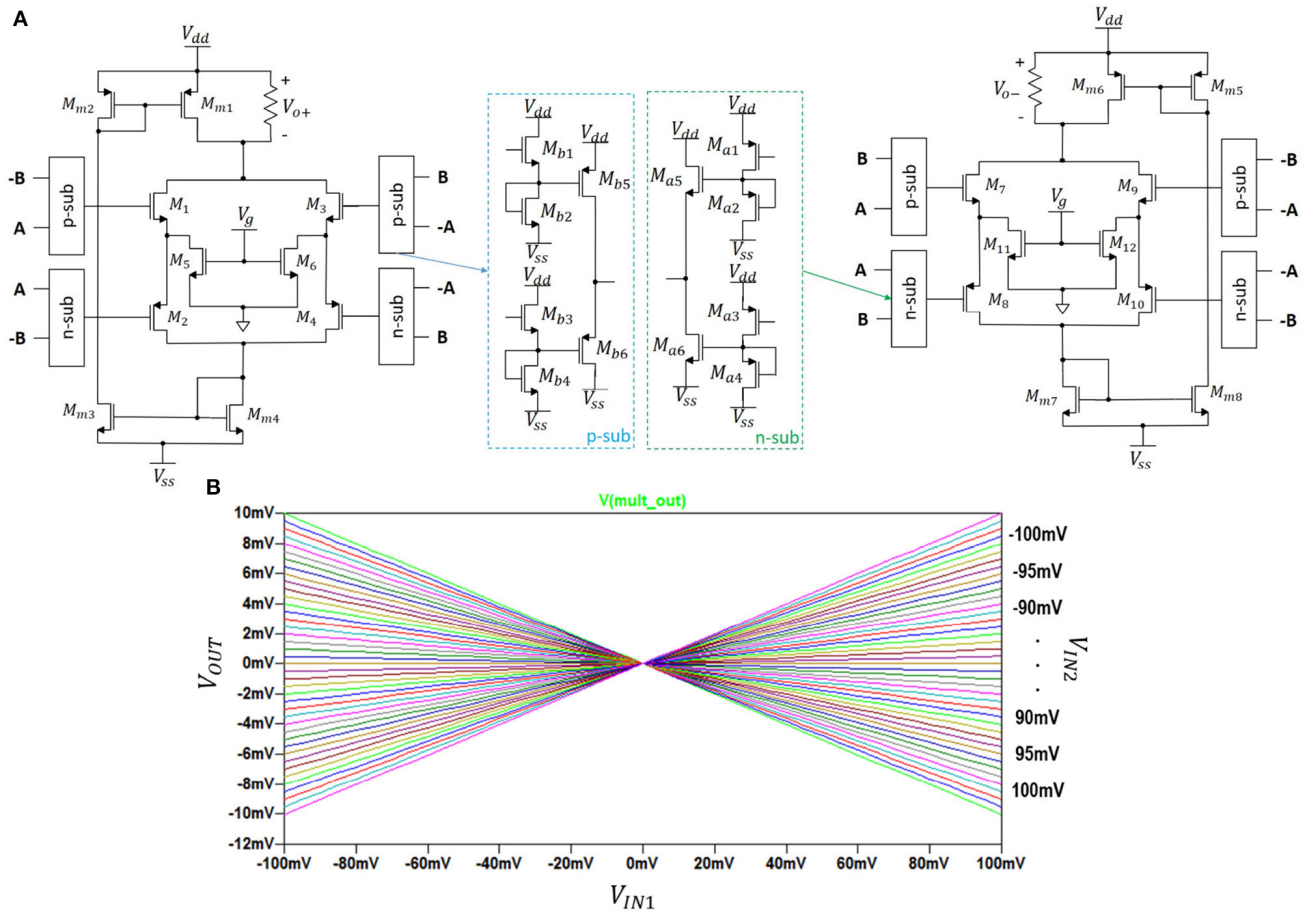
**(A)** The analog 4-quadrant multiplier with *V*_*g*_ = 1.5*V*; *R* = 1*kΩ*, and voltage range ±0.5*V*. **(B)** its output.

The circuit schematic (Li, 2000) of the multiplier is shown in two parts in Figures 3A,B. Its DC transfer characteristics is shown in Figure 3.

In Figure 3A, the left-hand side transistors *M*_1_ to *M*_6_ constitute an analog voltage square circuit and a symmetric complementary push-pull source follower (Li, 2000). Transistors *M*_5_ and *M*_6_ are in linear mode of operation with drain currents *I*_*D*5_ and *I*_*D*6_ and the symmetric output currents *I*_*o*+_ and *I*_*o*−_ as:


(1) $$\begin{array}{r} I_{D5}=\beta [(V_{GS5}-V_{Tn})V_{DS5}-\frac{1}{2}V_{DS5}^{2}], \end{array}$$

(2) $$\begin{array}{r} I_{D6}=\beta [(V_{GS6}-V_{Tn})V_{DS6}-\frac{1}{2}V_{DS6}^{2}], \end{array}$$

(3) $$\begin{array}{r} I_{o+}=I_{D5}+I_{D6}=-\beta {\left(A+B\right)}^{2}, \end{array}$$

(4) $$\begin{array}{r} I_{o-}=I_{D11}+I_{D12}=-\beta {\left(A-B\right)}^{2}. \end{array}$$

where $\beta =\mu_{n}C_{ox}{\left(\frac{W}{L}\right)}_{n}$. The difference of the above two output currents is proportional to the multiplication of the input voltages *A* and *B*:


(5) $$\begin{array}{r} I_{o}=I_{o+}-I_{o-}=-4\beta AB. \end{array}$$

#### 2.2.4. Operational Amplifier and Memory Units

**Figure 4:**
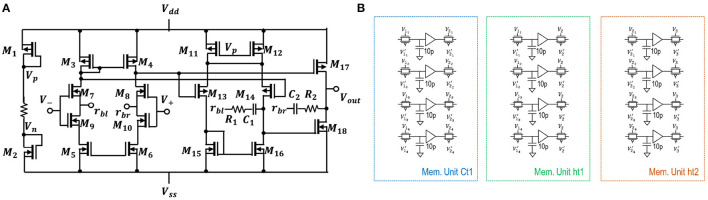
**(A)** Three-stage operational amplifier; **(B)** The illustration of capacitive memory units voltage buffer, and pass transistor switches used in the proposed LSTM design. The transistors has a W/L ratio 45 μm / 0.18 μm.

Figure 4A represents a low-power operational amplifier circuit utilised in voltage-driven LSTM. Its transistor-level implementation is described in section 4 of the Supplementary Material. Memory unit shown in Figure 4B holds values of the state *C*_*t*−1_ and output *h*_*t*−1_ from previous time steps of LSTM. Inside circuitry of memory units and some of the control voltages used can be observed in the same Figure 1. The control voltages to the sample and hold, and pass transistor switches are varied for NP LSTM (problem 1) memory units. The transistors have W/L ratios of 45μm/0.18μm. The capacitance of each capacitors is 10 pF. When the look-back number is equal to two, i.e., for problems 1 and 2, the same Figure 1 circuit blocks are used. When there look-back number is more than two such as for Problem 3, two-stage memory units are required. This additional memory unit is required to store current cell states while being able to retrieve previous cell states. As only first layer of LSTM is affected, addition is required only for “Ct1” and “ht1.” More details on the operation of the memory unit are provided in section 4 of the Supplementary Material.

### 2.3. Inference

In the inference stage, the weights from the training stage are fixed, and the models evaluated for the given set of problems it was trained. The proposed LSTM configuration was tested with discrete as well as continuous memristance values on all three problems. Using the memristor (Yu et al., 2011), in case of continuous values, the *R*_*on*_ and *R*_*off*_ were set to 10 *kΩ* and 10 *MΩ*. In case of simulations with discrete values, hafnium oxide (*HfO*_2_) memristor (Li et al., 2018) having *R*_*on*_ and *R*_*off*_, the values of 1.1 *kΩ* and 10 *kΩ* were used based on hafnium oxide (*HfO*_2_) memristor having 1.1 *kΩ* and 10 *kΩ* were used. The discrete simulations are more realistic as memristors (Li et al., 2018), show only limited stable states, for example 68 levels in Li et al. (2018). The circuit simulations are performed using TEAM memristive device model and 180 nm CMOS SPICE models.

### 2.4. Selected Models

Since the first problem was already solved algorithm wise in Brownlee (2016), using a two levels, i.e., LSTM followed by fully-connected dense layer. The LSTM used hidden units giving out four outputs and dense layer that gives a single predicted value. The look back number was kept as two as shown in He et al. (2000). Given the discontinuities of the data in problem 1 is more than that in problem 2 and 3, look-back value of two was empirically found to be more optimal. The networks were trained with Adam optimiser (Kingma and Ba, 2014) using the default parameters in Keras (2018). Our simulations used a much higher number of epoch (i.e., 500) as opposed to 100 as performed in Brownlee (2016). The higher number of epochs were required to match with the range of weights suitable to be implemented in hardware, with a weight variation tolerance of ±1. This is essential to match up with limitations of voltage inputs that can be applied to crossbar.

## 3. Results and Discussions

### 3.1. System-Level Simulations Results

The Monte Carlo (MC) simulations were used to compare the average inference time, training and test metrics for LSTM architectures. The LSTM is compared also with the simple-RNN (S-RNN) implementation.

The simulation results for each problem are tabulated in Table 2. Root Mean Square Error (RMSE) provides information on the standard deviation of prediction errors. It is noted that the performance of train RMSE, need not correspond always to test RMSE. On problem 3, however, the RMSE for the test and train correlate better than in problem 1 and 2, most probably due to variations captured train covers the range of variations in test. In this table, it also includes the number of parameters (weights) used in each architecture. Table 2 results can be summarised as follows. We can see from three cases that the conventional LSTM (NP) is better than the simple RNN. However, NP LSTM itself is not the best among its variations in terms of test scores. In fact, for each problem we have three different LSTM architectures as best-performing in test-score columns.

**Table 2:** The overall system comparison of LSTM architectures (*Inf. implies inference).

		**Problem 1**				**Problem 2**				**Problem 3**			
**#**	**RNNs**	**Test** **RMSE**	**Train** **RMSE**	*Inf. **time** **(ms)**	**Para-** **meter** **count**	**Test** **RMSE**	**Train** **RMSE**	*Inf. **time** **(ms)**	**Para-** **meter** **count**	**Test** **accu-** **racy**	**Train** **accu-** **racy**	*Inf. **time** **(s)**	**Para-** **meter** **count**
1	NP	0.102	0.0437	1.16	101	0.0465	0.0427	1.17	101	0.925	0.936	1.25	101
2	Vanilla	0.101	0.0429	1.31	113	0.0457	0.0412	1.30	113	0.935	0.943	1.31	113
3	NOG	0.106	**0.0402**	1.37	85	0.0443	0.0345	1.39	85	**0.951**	**0.958**	1.12	85
4	NIG	0.104	0.0410	1.58	85	0.0445	0.0343	1.53	85	0.891	0.907	1.13	85
5	NFG	0.107	0.0462	1.68	85	0.0555	0.0475	1.74	85	0.891	0.907	1.17	85
6	NIAF	0.106	0.0423	1.85	113	0.0564	0.0412	1.87	113	0.936	0.945	1.31	113
7	NOAF	**0.097**	0.0424	2.05	113	0.0457	0.0400	2.15	113	0.920	0.932	1.32	113
8	FGR	0.110	0.0414	2.46	257	0.0456	**0.0331**	2.39	257	0.936	0.945	1.94	257
9	GRU	0.111	0.0403	1.19	77	**0.0439**	0.0345	1.10	77	0.898	0.913	1.06	77
10	S-RNN	0.113	0.0413	1.16	29	0.0551	0.0334	1.02	29	0.891	0.907	0.69	29

*Bold values indicate the best results*.

The architectures for analog-hardware implementation were chosen using these column results. It should be noted that parameters such as number of weights and inference time were not taken into account. In fact, they are primarily tabulated to showcase that in digital hardware these indicators play a great role.

In other words, in digital domain we choose one algorithm over others as it requires lower latency and less memory while yielding good prediction or accuracy scores. Whereas, in analog hardware using memristive crossbars, these indicators become irrelevant since each time-step cycle takes the same amount of time (regardless of the number of weight parameters) and there is no data transfer delay to-and-from memory (regardless of the number of parameters to store).

In the former case, this is because VMM operations happen instantly (accumulation of currents) and in parallel when using memristive crossbars. As for the second case, since memristors can hold their states there is no need for separate memory space and consequently no need of transferring to-and-from a memory space. In addition, memristors have very small on-chip footprint which further emphasises the advantage of analog-memristive crossbar circuits over the digital hardware when dealing with very large weight matrices.

### 3.2. Circuit-Level Inference Results

In this part, the best performing LSTM architectures from the Table 2 were chosen for implementation in hardware. They include following configurations: No peephole (NP) and No Output Activation Function (NOAF) to solve problem 1; Coupled Input and Forget Gate (GRU) to solve problem 2; and No Output Gate (NOG) to solve problem 3. Each hidden unit requires one sample and hold circuit in its memory units. The control signals ensure a sequential operation, resulting in reduced area on chip but with increased inference time. About 40 μs is required for evaluating through all the four LSTM hidden layers, and total inference time of 88 μs for prediction problem 1 and 2 on a single dataset. While for problem 3, it takes 6.38 ms to evaluate 152 time steps of predictions.

**Figure 5:**
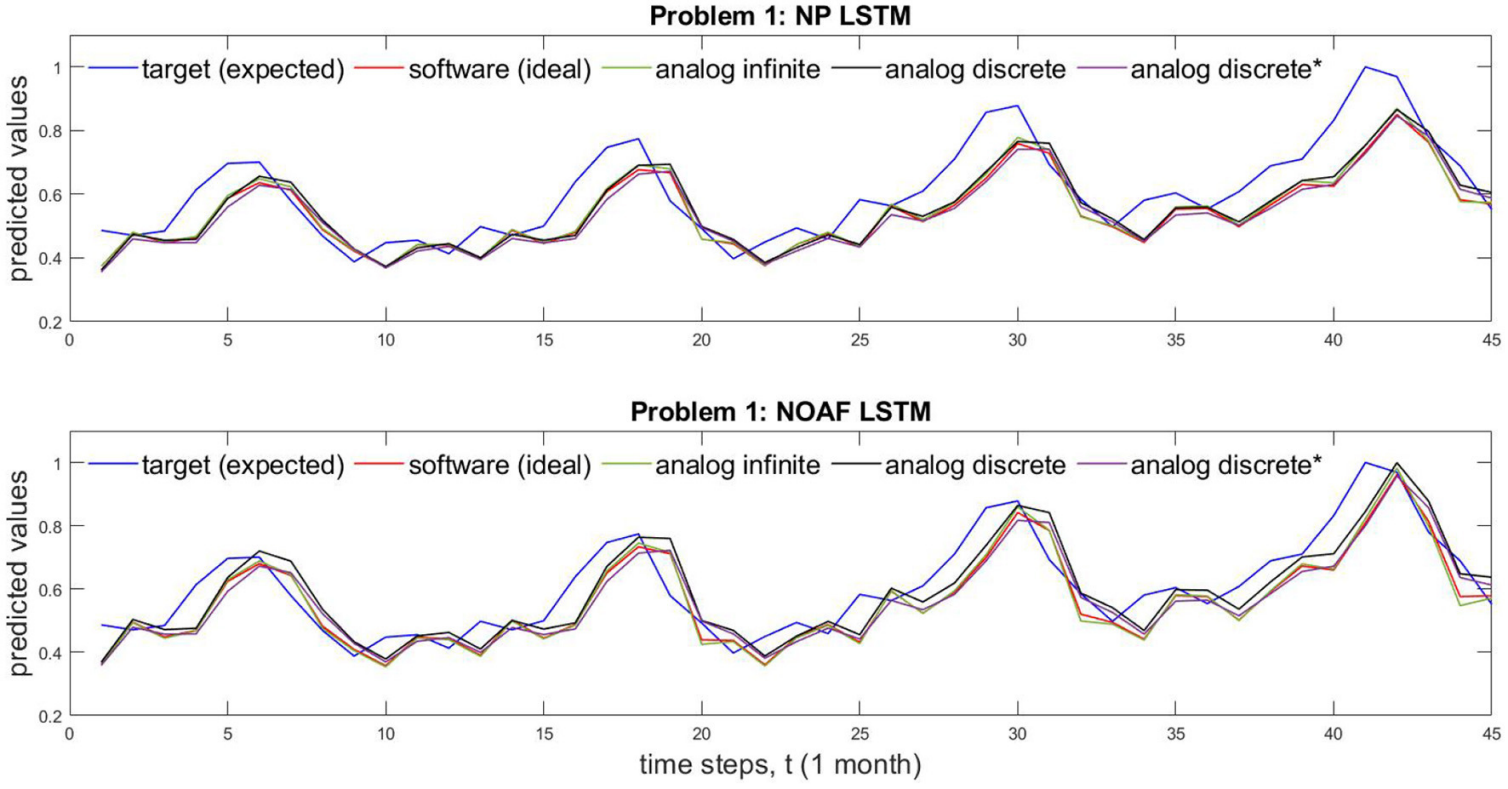
Comparison of NP and NOAF LSTM for both memristor with infinite and discrete states. For problem 1, time taken for inference is 3.96 ms taking 45 samples. *Represents impact of wire resistance.

**Figure 6:**
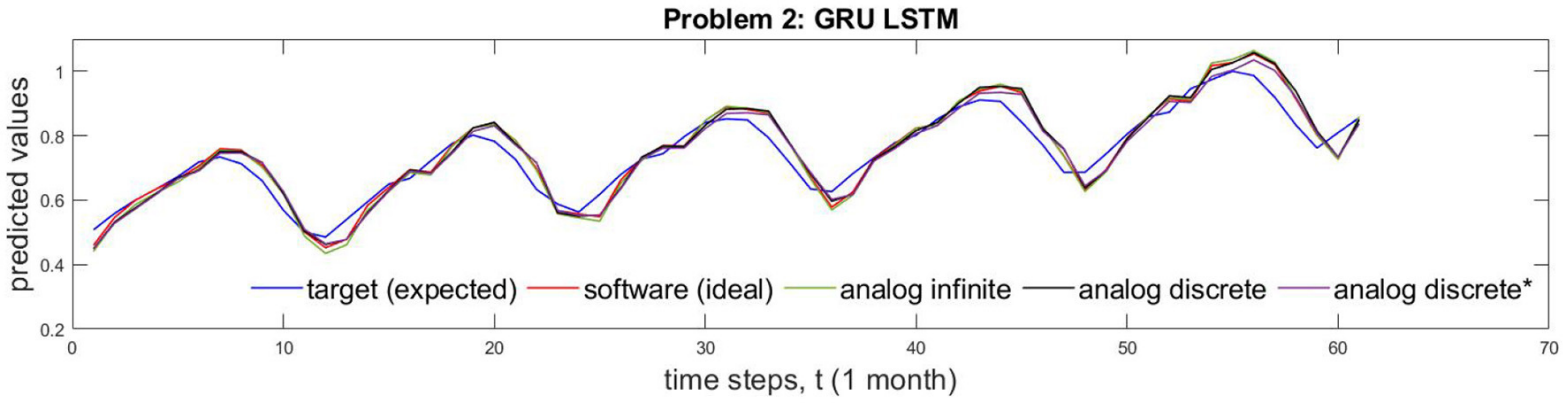
The comparison of GRU LSTM implementations of ideal algorithm, memtistors with infinite, and discrete states are shown for problem 2. *Indicates incorporation of crossbar wire resistances.

Based on data from Supplementary Table 2, Figure 5 shows different implementation results of Problem 1 which is solved by both NP and NOAF LSTMs. Figure 5 shows the comparison of circuit performance on infinite (continuous) and discrete conductance states. These two cases are juxtaposed with software implementation results and the desired target values. In addition, Figure 5 shows the comparison of analog discrete-memristance-state implementations with and without crossbar wire resistances. The numerical comparison of the waves in this figure is provided in Supplementary Table 2. In the same manner as described above, Problem 2 results, obtained by GRU LSTM, are plotted in Figure 6.

**Figure 7:**
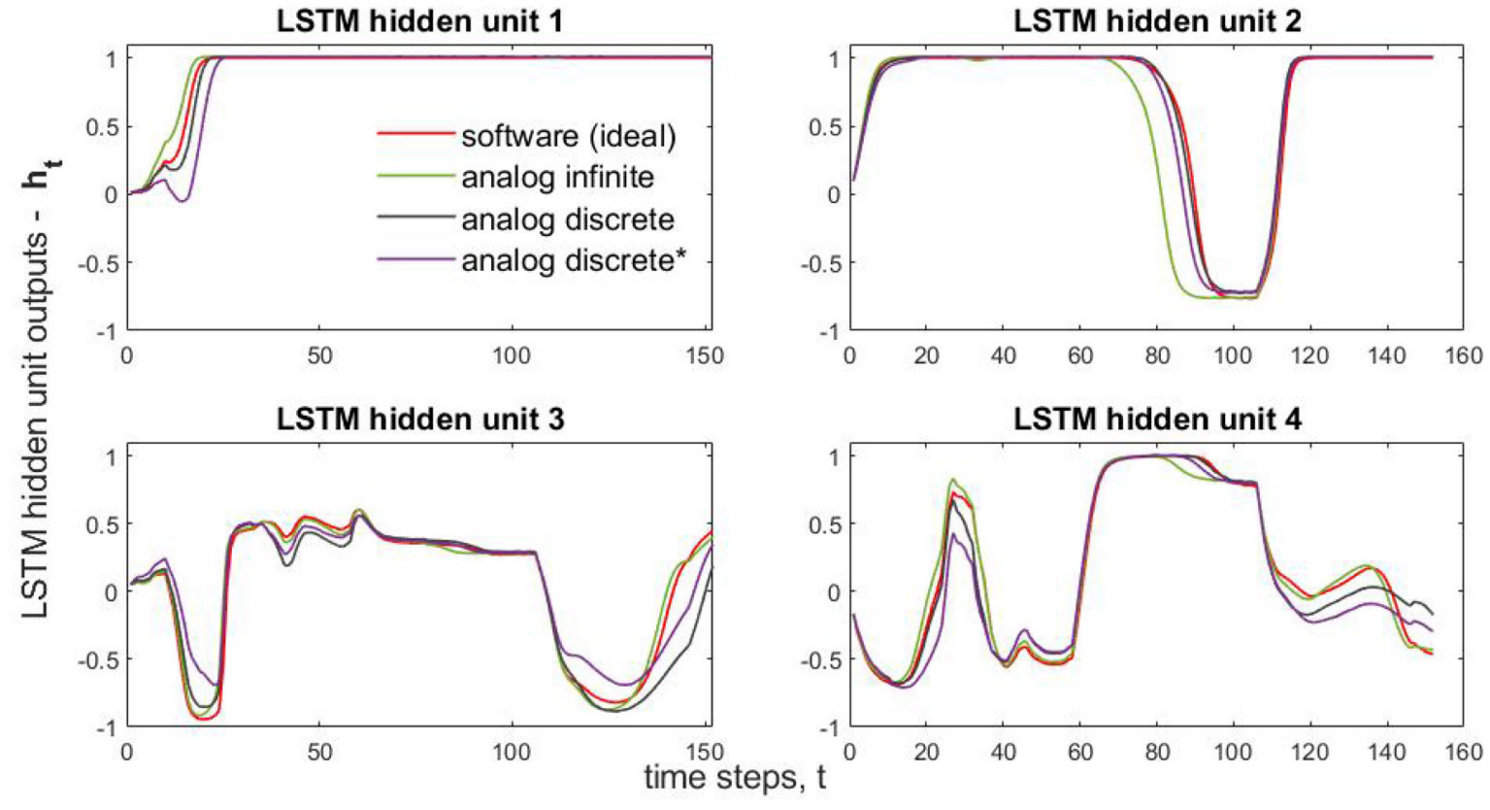
Classification of the Wafer 23: the comparison of NOG LSTM layer outputs for analog and algorithmic implementations. * indicates incorporation of crossbar wire resistances.

In Figure 7, we compare LSTM hidden unit values (four values per time step) obtained from different implementation ways. This obtained subplots only correspond to the classification of a single randomly chosen wafer (test wafer 23). That is they are obtained by running single dataset through the selected neural-network model. Due to the large volume of the testing data (6,164 datasets) and many (152) time-step operations in Problem 3, running through SPICE all of the test datasets is prohibitive. Therefore, we captured and plotted the intermediate results (four sub-results) of a single test case. As for Problem 3, solved by NOG LSTM, Additionally, Supplementary Table 2 presents comparisons of predicted and target values for ten different wafers.

### 3.3. Variability Analysis

**Figure 8:**
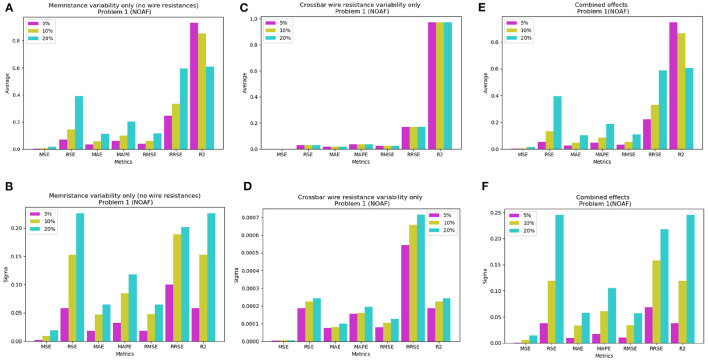
The mean performance on Problem 1 with NOAF LSTM (for 30 Monte Carlo SPICE circuit level simulations): an impact of 5, 10, and 20% Gaussian noise **(A)** in the memristors; **(B)** σ changes with variation of noise level in the memristors; **(C)** impact of noise level in the crossbar's resistance on averaged error; **(D)** σ changes with variation of noise level in the crossbar; **(E)** combined effect of **(A,C)**; **(F)** combined effect of **(B,D)**.

The widespread utilisation of memristive devices is hindered by a number of issues. These include thermal noise, retention, limited endurance, and other device imperfections. Although the inference stage runs at DC, the input of the crossbar arrays are time-dependent voltage pulses. Therefore, it increases memristors' susceptibility to the noise and cause conductance drift. Analysis in the section 3.2 did not take into account possible variations in the conductance of the memristors and in the crossbars. Random Gaussian noise with mean zero and σ of (5, 10, 20) percentages are added to memristance to emulate and study impact of variability on LSTM performance. The experiments are performed with 30 Monte Carlo simulations that results in degradation of performance (see Supplementary Table 4). The impact of wire resistance is also studied by adding memristor offsets. Their effect can be minimal if using back-end-of-the-line (BEOL) process (Li et al., 2019) which can give as low as 0.3 Ω wire resistance values between memristors. Using this experimental knowledge, the variability analysis with only the effect of the wire resistances was done by (1) choosing the mean value of wire resistances as 0.3 Ω; (2) adding random Gaussian noise and (3) running 30 Monte Carlo simulations. Figure 8 summarises these results for NOAF LSTM architecture, while additional results are shown in Supplementary Table 4 and Supplementary Figures 5, 6.

As it can be seen, there are seven metrics in total and six of them (i.e., MSE, RSE, MAE, MAPE, RMSE, RRSE) show similar dynamics to noise level changes in memristors. Particularly, error rate grows with the growth of the noise. In contrast, when noise introduced to the crossbar's wire resistance, the error rate for each metric is same regardless of noise level. This can be observed in Figure 8C. And therefore, as Figure 8E illustrates, the resulting combined effect from memristors' and crossbar's resistance variability has similar pattern as memristors' variability. Similar graphs for NP and GRU configurations of LSTM are presented in the Supplementary Materials. They exhibit similar behavior as NOAF architecture.

For additional results, the Supplementary Tables 2, 3 shows a singular case of 20% crossbar wire resistance variability was used. Finally, the combined effect of both non-idealities was performed and the results are shown in Supplementary Table 4. For this case each memristor's resistance was added as before with Gaussian noise σ = (5, 10, 20) percentages and each crossbar wire's resistance was added Gaussian noise with σ = 20 percentage. For the final two sets of variability simulations, the input voltages were scaled up to ±0.2 Volts to overcome the voltage drops caused by the wire resistances. This voltage range was chosen, because it does not disturb the conductances of the memristors (Li et al., 2018).

### 3.4. Area and Power Consumption

Power consumption and chip area calculations for the implemented architectures in this work are tabulated in Table 3. The area column lists the total area including the corresponding LSTM layer, dense layer, and the memory units inside them. The power consumption column only lists the maximum possible power needed in LSTM layer excluding the power consumed by memory units. This is done to keep consistent comparisons since depending on a problem different number of memory units are required and each of them can store different maximum possible values. However, in this work, the maximum possible power consumption by a memory unit (containing four sample and hold circuits) is 40.9 mW. This is more probable to happen when solving problem 3. The memristor is a non-volatile device with nanoscale size and therefore it does not significantly affect the total area and power consumption.

**Table 3:** Area and power consumption comparison.

**#**	**LSTM** **layer**	**Area** **(μm^2^)**	**Power** **(mW)**	**Input** **Range (V)**	**Roff(Ω)** **/Ron(Ω)**	**Which** **problem**
1	NP	143,090	233.35	[−0.2, 0.2]	10k/1.1k	Problem 1
2	NOAF	140,145	216.15	[−0.1, 0.1]	10k/1.1k	Problem 1
3	GRU	129,314	202.97	[−0.2, 0.2]	10k/1.1k	Problem 2
4	NOG	136,766	176.03	[−0.2, 0.2]	10k/1.1k	Problem 3

In addition, it is important to note that the power consumption calculation for the NOAF architecture used input range of ±0.1 V, because the lack of output activation function forces the output of the LSTM layer go beyond 0.2 V when using input range of ±0.2 V. Voltages higher 0.2 V in magnitude would disturb the memristor states in both LSTM and Dense layers. However, during the variability simulations involving crossbar wires, input voltages were increased to the range of ±0.2 V for NOAF architecture. This, however, did not result in the case where LSTM layer outputs going beyond 0.2 V in magnitude, because in realistic case all the inputs to the network would not equal 0.2 V at the same time and during the 2-time step processing cell state is not accumulated much.

## 4. Conclusion

To conclude, three practical problems were used in system-level and circuit-level simulations for demonstrating the use of proposed analog LSTM system for inference. Such system can be incorporated into sensors thereby allowing distributed analog computing for prediction and industrial information processing applications in real-time settings. Four different LSTM architectures, NP, NOAF, GRU, and NOG, were analysed with analog hardware using SPICE. The variability tests was performed by circuit simulations incorporating memristance variability, and crossbar wire resistance variability. The system was benchmarked for robustness to variability using 5, 10, and 20% of the mean values to generate Gaussian noises. The accuracy of the obtained results and the tolerance to circuit errors can be further increased by conducting more training epochs and consideration of hardware issues.

## Data Availability Statement

Publicly available datasets were analysed in this study. This data can be found here: http://www.timeseriesclassification.com/description.php?Dataset=Wafer.

## Author Contributions

KA performed the circuit analysis and experiments. KS contributed to the design of LSTM architecture and performing literature. AJ designed the overall system and circuit blocks. All authors took part in the writing.

## Conflict of Interest

The authors declare that the research was conducted in the absence of any commercial or financial relationships that could be construed as a potential conflict of interest.

## Publisher's Note

All claims expressed in this article are solely those of the authors and do not necessarily represent those of their affiliated organizations, or those of the publisher, the editors and the reviewers. Any product that may be evaluated in this article, or claim that may be made by its manufacturer, is not guaranteed or endorsed by the publisher.

## Footnotes

^1^This research extends the thesis (Adam, 2018) taking into account a range of practical variability of circuits. Further, the circuit blocks in this paper have been redesigned and further optimised for power and area improvements.
