# Supplementary material for: Generalised Analog LSTMs Recurrent Modules for Neural Computing
Source: Front Comput Neurosci. 2021 Sep 28;15:705050. doi: 10.3389/fncom.2021.705050 (PMC8506007; doi:10.3389/fncom.2021.705050)
Supplement: Supplementary file 1 [file Data_Sheet_1.pdf]

Supplementary Material

1 ACRONYMS

MAE Mean Absolute Error

MAPE Mean Absolute Percentage Error

MSE Mean Squared Error

RMSE Root Mean Squared Error

RRSE Root Relative Squared Error

RSE Residual Standard Error

R2 R-squared

GRU Gated Recurrent Unit

NIAF No input activation function

NIG No input gate

NOAF No output activation function

NOG No output gate

NP No peephole

S2T Software results with respect to the target values

A2T Analog results with respect to the target values

A2S Analog with respect to software results

AD2T Analog results with respect to the target values using discrete memristor levels

AD2T* Analog results with respect to the target values using discrete memristor levels and considering crossbar resistances

MC Monte Carlo

2 BACKGROUND

There is no control over the information that is fed into and out of a simple RNN cell. Whereas, an LSTM RNN cell has control over what is forgotten, what is fed into, and what is outputted in a cell. This is achieved through: forget, input, and output gated neural networks which utilize sigmoid activation functions. The sigmoid function changes smoothly and ranges between zero and one. When a gate's output is one, it allows passing all the current stage information to the next stage using Hadamard multiplication operation. Similarly, when it is zero, the gate does not allow to pass any information to the next stage.

There are total of nine existing architectures of LSTM Greff et al. (2017). They can be classified into six categories: 1) Vanilla; 2) No Peepholes (NP); 3) Full Gate Recurrence (FGR); 4) Coupled Input and Forgte Gate (CIFG); 5) with a Linear Activation Function; and 6) with a Constant Gate of Unity.

2.0.1 No peephole (NP) LSTM

From Figure S1, it can be seen that the structure of all three gates are the same: shared input vector which is the concatenation of network input value x_t at the current time step, bias, and the output value h_{t-1}, which is the output of the previous time-step LSTM cell; and the same activation functions and Hadamard multiplication

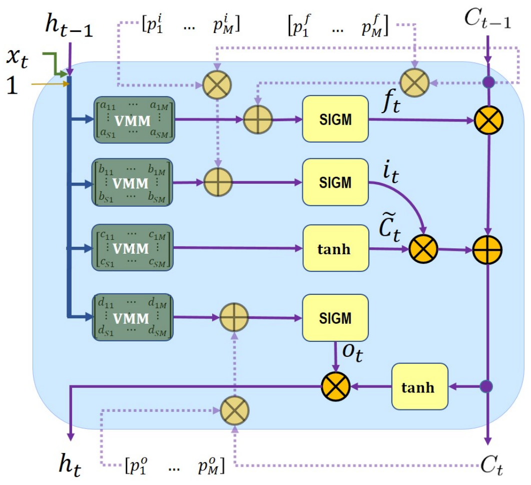

Figure S1: LSTM cell architecture. Letters sub-scripted with t or t-1 represent vectors of size M, except x_t which has size N. Bias is a scalar which is equal to unity. Dashed lines correspond to peephole connections in Vanilla LSTM.

units. They differ only in different values of weights for the input vector $[x_t, h_{t-1}, 1]$ as it can be seen below in mathematical form Zaremba et al. (2014):

$$f_t = \sigma(W_f x_t + U_f h_{t-1} + b_f), \tag{S1}$$

$$i_t = \sigma(W_i x_t + U_i h_{t-1} + b_i), \tag{S2}$$

$$o_t = \sigma(W_o x_t + U_o h_{t-1} + b_o). \tag{S3}$$

The cell state C_t of an LSTM cell is updated by forgetting some portion of the old cell state C_{t-1} and adding some portion of the candidate value $\tilde{C}_t$, which has the same structure as the gates, except with different activation function:

$$\tilde{C}_t = tanh(W_{\tilde{C}} x_t + U_{\tilde{C}} h_{t-1} + b_{\tilde{C}}), \tag{S4}$$

$$C_t = i_t \odot \tilde{C}_t + f_t \odot C_{t-1}. \tag{S5}$$

Finally, the current output of the LSTM cell is the some portion of the filtered cell state C_t:

$$h_t = o_t \odot tanh(C_t). \tag{S6}$$

Note that the equations S1-S6 correspond to the case of a single LSTM hidden unit. In practice, LSTMs are used with larger weight matrices meaning that the size of LSTM hidden layer is much larger. Also note that Keras library of Tensorflow uses the version of LSTM that was presented above Documentation (2018) which is also called No Peephole (NP) LSTM.

2.0.2 Vanilla LSTM

Hochreiter and Schmidhuber Hochreiter and Schmidhuber (1997) invented the very first LSTM. Later the original LSTM evolved into the most common architecture in Graves and Schmidhuber (2005) known as vanilla LSTM Greff et al. (2017). Figure S1 shows the detailed diagram of the vanilla LSTM layer. Vanilla LSTM differs from the standard LSTM used in Keras library Documentation (2018) by additional weighted connections known as peepholes. In Fig. S1, peephole weight vectors, half-transparent dashed lines, and math elements (multiplication and addition) are extra additions that separate the standard LSTM from the vanilla LSTM.

The vanilla LSTM shown in Figure S1 can be described in concise mathematical forms below Greff et al. (2017), where Q having size of S-by-M is the concatenation of input weight matrix W, recurrent weight matrix R, and

bias weight vector b along row axis; and q is the input vector of size 1-by-S:

$$f_t = \sigma(q_t Q_f + C_{t-1} \odot p_f), \tag{S7}$$

$$i_t = \sigma(q_t Q_i + C_{t-1} \odot p_i), \tag{S8}$$

$$\tilde{C}_t = tanh(q_t Q_{\tilde{C}}), \tag{S9}$$

$$C_t = i_t \odot \tilde{C}_t + f_t \odot C_{t-1}, \tag{S10}$$

$$o_t = \sigma(q_t Q_o + C_t \odot p_o), \tag{S11}$$

$$h_t = o_t \odot tanh(C_t), \tag{S12}$$

where p_f, p_i, and p_o represent peephole weight vectors.

2.0.3 Full Gate Recurrence (FGR) LSTM

FGR is the most complex architecture of LSTM. As its name suggests, Full Gate Recurrence LSTM is featured by recurrent connections between its all gates. FGR is basically vanilla LSTM (except peephole weights of output gate are element-wise multiplied to previous cell state vector) plus the new recurrent connections among the gates. It adds to the weight matrix Q of each gate additional $3M$-by-M recurrent weight matrix. Mathematically in detail, it can be described as following Greff et al. (2017):

$$f_t = \sigma(q_t Q_f + i_{t-1} R_{if} + f_{t-1} R_{ff} + o_{t-1} R_{of} + C_{t-1} \odot p_f), \tag{S13}$$

$$i_t = \sigma(q_t Q_i + i_{t-1} R_{ii} + f_{t-1} R_{fi} + o_{t-1} R_{oi} + C_{t-1} \odot p_i), \tag{S14}$$

$$o_t = \sigma(q_t Q_o + i_{t-1} R_{io} + f_{t-1} R_{fo} + o_{t-1} R_{oo} + C_{t-1} \odot p_o), \tag{S15}$$

2.0.4 Coupled Input and Forget Gate (CIFG) or GRU

CIFG architecture of LSTM is more known as GRU Cho et al. (2014). Again, its name (Coupled Input and Forget Gate) explains itself: $f_t = 1 - i_t$. However, other than that, 1) there are also no peephole connections and no output activation function; 2) candidate cell state's recurrent inputs are filtered through output gate before being multiplied with recurrent weight matrix; and 3) cell state and cell output are combined together. The differences can be easier to see in mathematical equations:

$$\tilde{C}_t = tanh(x_t W_{\tilde{C}} + (h_{t-1} \odot o_t) R_{\tilde{C}} + b_{\tilde{C}}), \tag{S16}$$

$$h_t = f_t \odot \tilde{C}_t + (1 - f_t) \odot h_{t-1}, \tag{S17}$$

2.0.5 Other LSTM architectures

The next category falls into LSTM architectures with a linear activation function either in a) candidate cell state generation stage or in b) output gate stage. From the vanilla LSTM equations, only equations S9 and S12 change. In architecture a), known as No Input Activation Function (NIAF), equation S9 becomes $\tilde{C}_t = (q_t Q_{\tilde{C}})$ In architecture b), known as No Output Activation Function (NOAF), equation S12 becomes $h_t = o_t \odot C_t$. The last category includes LSTM architectures which have a constant gate of unity in one of its gates and everything else is the same as in the vanilla LSTM. Then there are three different such architectures: a) No Input Gate (NIG): $i_t = 1$; b) No Forget Gate (NFG): $f_t = 1$; and c) No Output Gate (NOG): $o_t = 1$.

2.1 Traditional hardware implementation of LSTM

RNNs, and LSTM in particular, are characterized by massive parallelism and high complexity during learning and inference operations. These complicate execution on conventional computers which mainly use CPUs. Therefore, preference is given to customized hardware like GPU, FPGA and ASIC. Analysis of the available in the market

hardware solutions has shown, that FPGA is more efficient than CPU and GPU. In addition, evaluation results demonstrated little gap between ASIC and FPGA performances Nurvitadhi et al. (2016). A survey by Wang et al. (2018) provides a comparison table of FPGA-based LSTM implementation and discusses advantages and disadvantages of it. In particular, reconfigurability of FPGA brings flexibility in design changes, but also found to be time and resource hungry process. In general power consumption of FPGA-LSTM lies in the range between 20-41W.

2.2 Memristive Crossbar Arrays

The idea of two-dimensional resistive crossbar arrays was first proposed in 1961 by Steinbuch Steinbuch (1961). Memristors in crossbar configurations has a wide range of applications in neural network designs. Figure S2 shows the implementation of a single layer of neural network using memristive crossbar. The inputs x_1, x_2, and x_3 are represented by voltage sources v_1, v_2, and v_3 in the circuit. The weights from w_{11} to w_{33} correspond to the memristor conductance from G_{11} to G_{33} in the same order. Finally, as expected, the outputs y_1, y_2, and y_3 correspond to currents i_1, i_2, and i_3. In practical implementations, the currents go to virtual grounds created using operational amplifiers. Note that here linear activation functions in the output layer were used for simplicity. Basically, the crossbar executes vector-matrix multiplication (VMM) operation in parallel at high speeds reaching from 1,000 up to 10,000 times speed-efficiency Hu et al. (2016). Another advantage of the memristive crossbar is that there is no need to store weight values somewhere else – training of the weights happens in-place. In case of conventional computers, increase in neural network size creates bottleneck during data transfer (between CPU and memory) and processing (sequential execution of VMM by processing units).

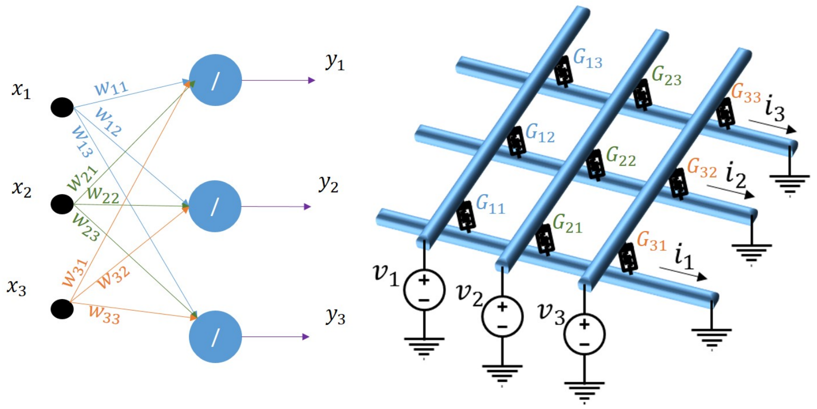

Figure S2: Simple neural network (left) and its crossbar implementation (right).

2.3 Memristive hardware implementation of LSTM

In the recent work Gokmen et al. (2018), the memristive LSTM was simulated using their own built software tool (written in C++) rather than on circuit simulator such as SPICE, excluding the non-idealities associated with the dynamic behaviour of memristors. The non-linear function units (NLFs), which are digital blocks, are used to implement all the mathematical operations required for implementing LSTM except for VMM. The VMM itself is implemented using memristive crossbars.

In another recent implementation, a successful on-chip circuit for learning memristor crossbar for dot-product computations was implemented in the digital domain using a co-processor Li et al. (2019). The existing implementations make use of a mixed signal design approach to perform learning and VMM computations for each of the gated neural networks. One more complete solution of memristive LSTM (MLSTM) hardware platform was presented by Wen et al.Wen et al. (2019). Their MLSTM uses circulatory approach where instead of M cells in a layer, a single LSTM unit is used M times. Their memristor crossbar in LSTM unit was trained externally using Jo et al. modelJo et al. (2010). Authors also compared the maximum power consumption of a single memristor synapse which is equal to 5.6 μW to that of a CMOS-based synapse which is around 50mW.

In the previous work Smagulova et al. (2018b), a purely analog implementation of memristive LSTM with 0.18 μm CMOS technology is proposed. However, this work does not provide a full circuit simulation of the whole system for solving a particular machine learning problem. As opposed to Smagulova et al. (2018b), a work by Smagulova et al. (2018a) offered full current-based CMOS-memristor analog circuit design of an LSTM layer for time-series prediction problem from Brownlee (2016). In Adam et al. (2018a), we introduced voltage-based circuit parts due to their ability to produce accurate and predictable outputs which are essential in forecasting problems.

In addition, this work Adam et al. (2018a) featured control circuit and memory units to incorporate two time-step operation of LSTM.As for our work in Adam et al. (2018b), using the voltage-based LSTM, we were also able to solve a classification problem data (2018) which involved long time-step recurrences. Even if it is a classification problem, the many time-step operations degrade the final time-step circuit output. Therefore, even in this case it is required to have high-accuracy circuit components.

3 SELECTED MODELS

Former Section 4 became Section 3

Since the first problem was already solved in Brownlee (2016), the model selection process was minimal. To solve the problem they used a two-layer neural network consisting of LSTM and Dense (fully-connected) layers. The LSTM layer in the network contains four (a not too small and not too large value) hidden units and the Dense layer contains a single unit which squashes the four outputs of the LSTM layer into a single output – prediction value. Another parameter is a look-back number or the number of recurrent operations before obtaining the last time step output. It was chosen to be equal to two, which was based on He et al. (2000). It was also verified empirically: for the same number of hidden units, it gave a better result for a look-back value of two than for values of one and three. One might question that if LSTM is good at learning long patterns of data through time then why it did not do well for a look-back value of three. It may be due to the lack of extra features or there is a bad pattern in the data. Also, note that problem 2 data follows a better pattern than that of problem 1; and problem 3 has similar shapes for the same class data plots. One should keep in mind these observations when looking at the system-level simulation results. As for the training of the network, it was trained using mean square error loss function and Adam optimizer Kingma and Ba (2014) with default parameters Keras (2018). However, instead of 100 epochs as in Brownlee (2016), an epoch size of 500 was chosen while keeping a batch size of one. It was done to obtain approximately the same performance results as in the former case while having weight constraints between ± 1. This is needed for keeping input voltages into memristive crossbar arrays small enough for a given range of memristances, $[R_{on}, R_{off}]$.

4 CIRCUIT BLOCKS AND CONTROL SIGNALS

We propose a hardware implementation of a voltage-based base LSTM network architecture for a set of time-series forecasting and classification problems. Figure 1 in the Manuscript shows its overall implementation. It is comprised of several circuit blocks for implementation of computations within LSTM cell:

- Crossbar array for implementation of the dot-product operation;
- Activation function circuit for implementation of sigmoid and hyperbolic tangent functions;
- Four-quadrant multiplier;
- Voltage-Adder.

Besides, it includes operational amplifier (OpAmps) circuits for sensing output current of the crossbar array and converting it to voltage values. In addition, OpAmps are used for adjustment of voltage levels of the input signals to the crossbar array, activation function circuits. The circuit design also includes pass transistor switches to implement the LSTM in sequential manner. To store intermediate values, LSTM design has memory units comprised of sample and hold circuits. They are used to store the previous time-step cell state values and the previous time-step outputs of LSTM hidden units The values of the utilized capacitors are 10pF. All of the circuits were designed using 180nm

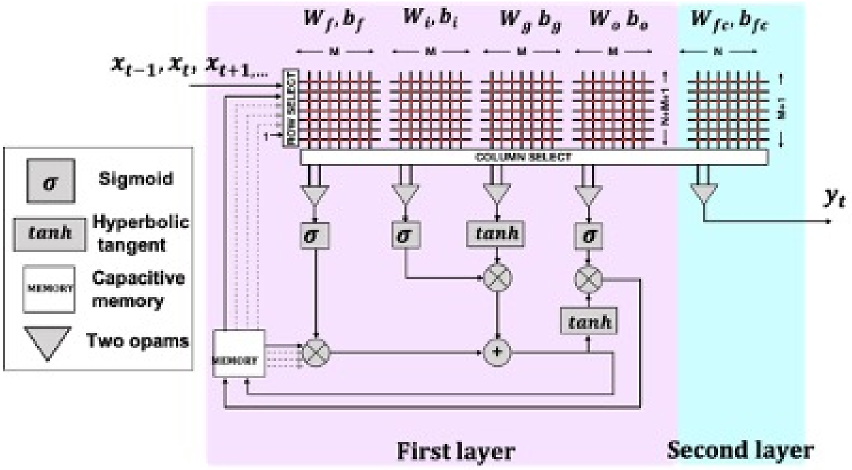

Figure S3: Overall schematic of the LSTM unit.

Table S1. Analog LSTM circuit blocks and their parameters

**Circuit Block**	**Parameters**
Operational Amplifier	M1=3.96um/0.36um; M2=1.8u/0.36u; M3 = M4 = 3.96u/0.36u; M5 = M6=M15=M16 1.8u/0.36u;<br>M7 = M8 = M9 = M10 = M11= M12 = 3.6u/0.36u; M13 = M14 = 12u/0.36u;<br>C1 = 5fF; C2=10fF; R1=124 Ohm; R2= 1.14KOhm, R3=0.5KOhm
Four-quadrant multiplier	M1 = M2 = M3 = M4 = M7 = M8 =M9 =M10 = 7.2u/0.36u;<br>M5 = M6 = M11 = M12 = 199.98u/0.36u; Mm1 = Mm2= Mm3 =Mm4 = 1.26u/2.4u;<br>Ma1 = Ma2 = Ma3 = Ma4 = 0.4u/0.36u; Ma5 = Ma6 = 0.84u/0.36u;<br>Mb1 = Mb2 = Mb3 = Mb4 = 0.4u/0.36u; Mb5 = Mb6 = 0.72u/0.36u;
Activation function	N1 = N2 = 0.9u/0.18u; R =2KOhm.

TSMC CMOS technology. The schematics of the circuit blocks utilized for implementation of the generalized LSTM are provided in Section 2.2 of the Manuscript. In this work, area was estimated based on the number and size of components. Power consumption was evaluated based on power consumption of the components in each circuit block. Here, Table S1 represents the parameters of the transistor-level realization of certain circuit blocks. This base LSTM architecture is then used to build other LSTM architectures that are specific problem oriented.

In order to get results for Problems 1 and 2, the normalized input data are supplied sequentially to the input of the memristor crossbar array. At one time step, only one input value is fed. Prior being processed by LSTM circuit it is downscaled between (-0.1;0.1)V for implementation of NP and NOG configurations and between (-0.2;0.2)V for implementation GRU and NOAF configurations. The number of hidden units in LSTM is chosen to be 4. As for the control voltages, they ensure that each LSTM cell output is obtained in sequential manner. It takes 40 us to obtain all four LSTM hidden unit outputs at a current time step and total of 88 us to predict the target value of a single dataset since look-back is equal to two. Therefore, each prediction is computed in two time-steps or 88us. Similarly, output is computed for problem 3 with the look-back equal to 152. Each weight value in the neural network was represented as a difference of conductance of two memristors located at two adjacent columns. Therefore, the number of columns in memristive LSTM doubles and equal to [6,32]. At the first time step, the output currents are sensed by two OpAmps. The first OpAmp performs current subtraction. The second amplifier converts computed current to voltage value which is passed to the corresponding activation function circuit. This process is repeated four times which correspond to the number of hidden units. Since four hidden units are used in the network configuration, there are four sample and hold circuits in each memory unit. Depending on the LSTM gate (e.g., f_t, o_t, i_t or g_t), output of the activation function circuit is passed to the certain four-quadrant multiplier circuit and so on. After first time step, the procedure is repeated required number of times in LSTM Layer 1 using control signal and pass transistor switches. Then, the final prediction is made at fully-connected Dense Layer 2 as shown in Figure S3.

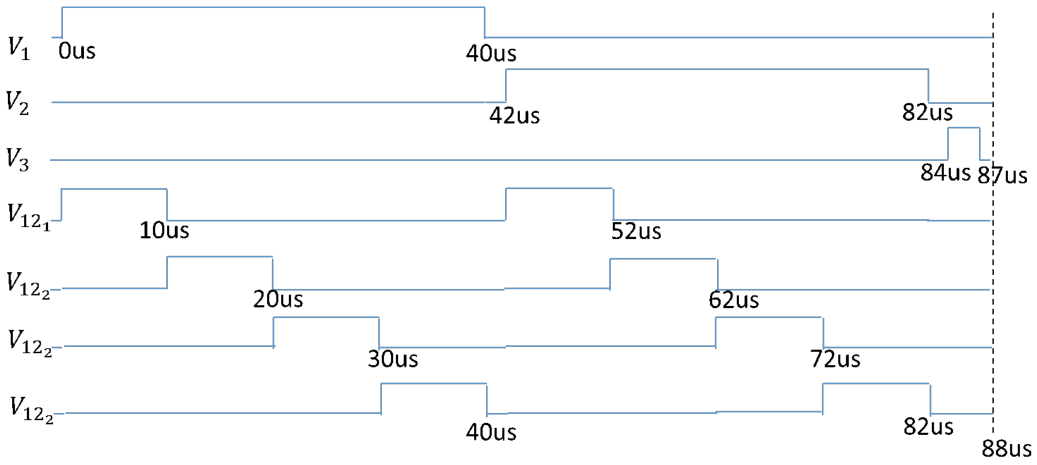

Figure S4: Control Voltage signals for voltage-based LSTM solving problem 1. Obtaining a single prediction value x_{t3} takes 88μs. Amplitude of the pulses is 1.8V. The complements of the control voltages have the same pattern, but amplitudes of -1.8V. Voltages V_{1_1} to V_{1_4}; and V_{2_1} to V_{2_4} are not shown in this figure, but they are first and second halves of signals V_{12_1} to V_{12_4}, respectively.

Figures 5-8 in the Manuscript compare the dataset values with the predicted values during system and circuit level implementation. Figure 5 illustrates predictions made for Problem 1. It consists of 45 values predicted during 3.96ms. Similarly prediction is made for Problem 2. Figure 8 shows values computed for Problem 3 at the LSTM Layer 1 before the final prediction is made at Dense Layer 2.

5 RESULTS

In this table S2 , 'S2T' means that the error metrics are calculated for software results with respect to the target values. It is the same for 'A2T' column except using analog results (obtained using infinite memristance states) instead of software results. Similarly, 'A2S' refers to metrics calculated when comparing the analog-obtained (using infinite memristance states) prediction values with respect to software-obtained prediction values. The last two columns – 'AD2T' and 'AD2T*' – refer to the analog implementation of LSTM with discrete memristance states. The * symbol indicates the usage of crossbar wire resistances. Here, 'AD2T*' basically lists the results for a singular case from the variability simulations which is discussed later in this section. In Table S2, R^2 scores of 0.975 and 0.980 can be observed for 'AD2T' and 'AD2T*' columns, respectively, for NP LSTM. The higher R^2 score in the second case is achieved, because its waveform is moved closer to the software-obtained waveform when incorporating the wires. The effect from the crossbar wire resistances was small and therefore a small shift in the waveform occurred. By chance this shift happened to be towards the software-obtained waveform. Similarly, this can be observed in NOAF LSTM, too, with R^2 scores of 0.945 and 0.971 for 'AD2T' and 'AD2T*' columns, respectively. However, in cases with GRU and NOG LSTMs R^2 scores degrade when simulating with crossbar wire resistances. In these cases, the shifts in waveforms happen to be away from the corresponding software-obtained waveforms. In addition, it can be observed in Table S2 that 'A2T' gives less errors than 'S2T' for Problem 1 (NP) and Problem 2 (NOAF) cases. This is due to the small errors circuit parts contributing at each stage. However, the final error can be lower or higher than that of 'S2T' case depending how the small non-idealities happen to work on our favor or not. An exaggerated analogy, but it is similar to adding non-linearity in a neural network. Here, our non-idealities play the role of non-linearity. In the same way as different non-linear functions can be good or bad for our neural network, the circuit non-idealities can decrease or increase the overall time-series prediction error of our circuit. This can be evidenced in Table III for Problem 2 (GRU) case where errors happen to be larger when solving it in analog domain.

In table S2, problem 3 software values correspond to hidden unit values at each time step; and the metrics were averaged. Yet, the final outputs of 10 randomly chosen test datasets are captured for different NOG LSTM

Table S2. Numerical Comparison Results of LSTM configurations (also illustrated in the Figures 9-11 in the paper). * indicates incorporation of crossbar wire resistances.

	Problem 1 (NP)					Problem 1 (NOAF)					Problem 2 (GRU)					Problem 3 (NOG)		
Metrics	S2T	A2T	A2S	AD2S	AD2S*	S2T	A2T	A2S	AD2S	AD2S*	S2T	A2T	A2S	AD2S	AD2S*	A2S	AD2S	AD2S*
MSE	1.01E-2	9.15E-3	6.75E-5	3.70E-4	2.65E-4	7.70E-3	7.81E-3	7.56E-5	1.25E-3	5.59E-4	1.86E-3	2.21E-3	6.61E-5	1.14E-4	2.06E-4	2.45E-2	1.26E-2	1.98E-2
RSE	5.90E-1	5.24E-1	4.81E-3	2.51E-2	2.01E-2	3.53E-1	3.35E-1	3.57E-3	5.48E-2	2.93E-2	8.69E-2	9.43E-2	2.82E-3	5.16E-3	1.03E-2	6.93E-2	5.19E-2	1.05E-1
MAE	8.00E-2	7.73E-2	6.38E-3	1.53E-2	1.34E-2	7.17E-2	7.15E-2	6.18E-3	3.02E-2	1.93E-2	3.50E-2	3.96E-2	6.55E-3	8.52E-3	1.19E-2	5.37E-2	5.88E-2	7.83E-2
MAPE	1.24E-1	1.22E-1	1.06E-2	2.79E-2	2.53E-2	1.17E-1	1.18E-1	1.06E-2	5.40E-2	3.61E-2	4.78E-2	5.48E-2	1.02E-2	1.21E-2	1.62E-2	3.15E-1	5.39E-1	8.24E-1
RMSE	1.01E-1	9.57E-2	8.22E-3	1.92E-2	1.63E-2	8.77E-2	8.84E-2	8.70E-3	3.54E-2	2.36E-2	4.31E-2	4.70E-2	8.13E-3	1.07E-2	1.44E-2	1.20E-1	9.91E-2	1.39E-1
RRSE	7.68E-1	7.24E-1	6.93E-2	1.58E-1	1.42E-1	5.94E-1	5.79E-1	5.98E-2	2.34E-1	1.71E-1	2.95E-1	3.07E-1	5.31E-2	7.18E-2	1.01E-1	2.30E-1	2.07E-1	3.13E-1
R2	4.10E-1	4.76E-1	9.95E-1	9.75E-1	9.80E-1	6.47E-1	6.65E-1	9.96E-1	9.45E-1	9.71E-1	9.13E-1	9.06E-1	9.97E-1	9.95E-1	9.90E-1	9.31E-1	9.48E-1	8.95E-1

Table S3. Problem 3 target and prediction (NOG LSTM implementation) values. * indicates incorporation of crossbar wire resistances.

wafer test #	target (expected)	software (ideal)	analog infinite (mV)	analog discrete (mV)	analog discrete* (mV)
23	-0.50	-0.47	-42.2	-27.0	-24.9
47	0.50	0.49	46.9	29.8	24.2
7	-0.50	-0.51	-45.9	-34.0	-28.1
3	0.50	0.49	52.6	31.0	29.3
3838	-0.50	-0.28	-27.4	-36.6	-19.4
193	0.50	0.53	55.0	33.8	37.3
6157	-0.50	-0.47	-41.4	-9.94	-16.5
411	0.50	0.54	52.9	33.5	36.4
1534	-0.50	-0.32	-30.1	-39.4	-22.2
4507	0.50	0.42	31.3	26.8	27.9

implementations. They are listed in Table S3 along with the target (expected) values. Since this is a binary classification problem, the sign of the predicted values determined the correctness of classifications. Therefore, in Table S3, all implementations yielded 100% accuracy for classifying those 10 test wafers. However, the prediction values from the analog implementations when multiplied by 10 should ideally match the software-obtained values. As for the variability simulations in SPICE, we also restricted ourselves to run them on the same 10 test wafer datasets only. Each test wafer dataset goes through 30 Monte Carlo circuit simulations for a single set of variability simulations. Since this is a classification problem the results of the variability simulations are captured using accuracies for each wafer. That is the results tell in percentages how many times (out of 30) the same wafer dataset is classified correctly.

6 VARIABILITY ANALYSIS

The lifetime of memristor device depends on its switching and electrode materials, the value of read/write voltages and condition of the device. One of the common issues of memristive devices is stack-at-0 or stack-at-1 problems. It can be solved by retraining of neural network and reprogramming of the crossbar array. The results of the study of tolerance to resistive states variability are provided in this Section. The scores in tables S2 and S3 were obtained when considering no variability in the conductances o the memristors in the crossbars. Whereas, introducing variability by adding Gaussian noise to memristances of the memristors with the noise's standard deviation equal to 5%, 10%, and 20% and running 30 Monte Carlo simulations give an expected degrading performance results as observed in the left-hand side of Table S4.

In addition, there is a need to simulate the real effect of wire resistances in the crossbar arrays similar to the case with the memristance variability. Their effect can be minimal if using back-end-of-the-line (BEOL) process Li et al. (2019) which can give as low as 0.3 Ω wire resistance values between memristors. Using this experimental knowledge, the variability analysis with only the effect of the wire resistances was done by 1) choosing the mean value of wire resistances as 0.3 Ω; 2) adding Gaussian noise with the standard deviations equal to 5%, 10%, and 20% of the mean; and 3) running 30 Monte Carlo simulations. The results are shown in the right-hand side of Table S4. In tables S2 and S3, a singular case of 20% crossbar wire resistance variability was used. Finally, the combined

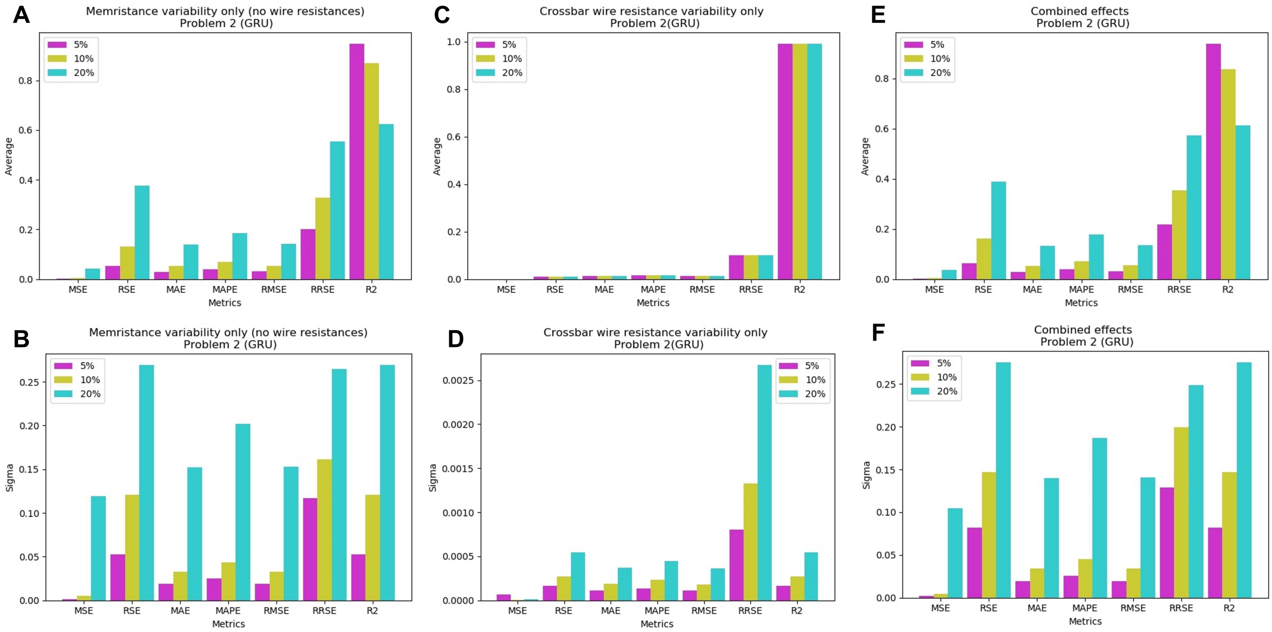

Figure S5: Average Performance results of 30 MC SPICE simulations with combined effects from both noisy memristor resistances and noisy crossbar wire resistances for Problem 2 (GRU).

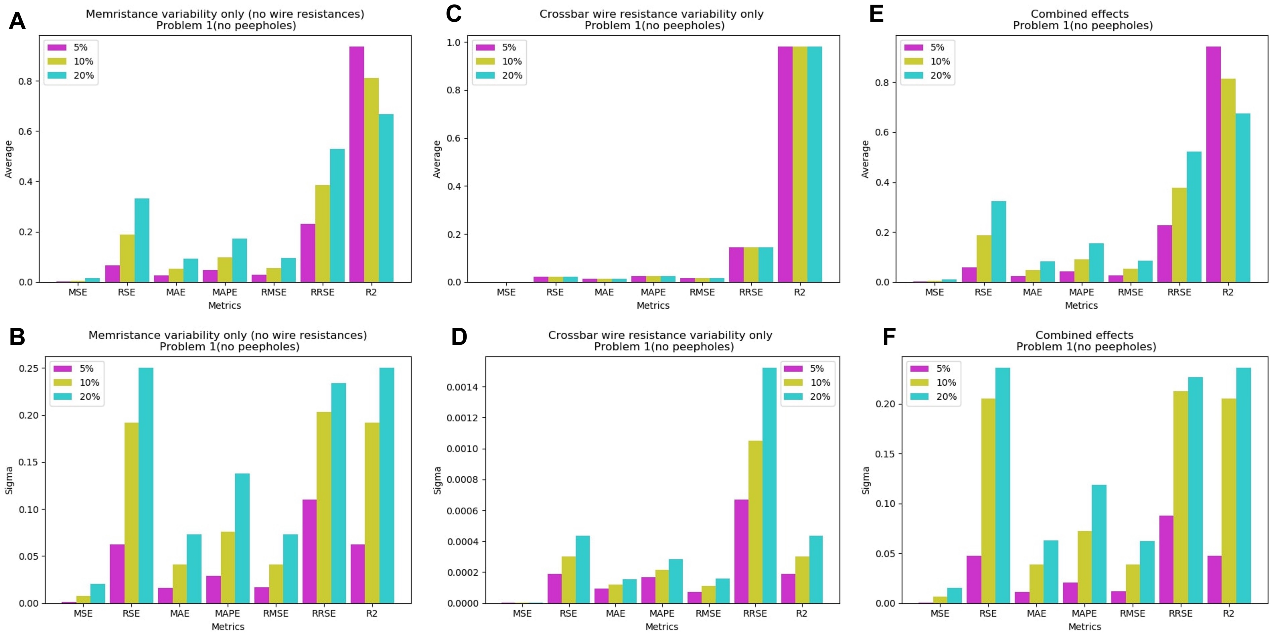

Figure S6: Average Performance results of 30 MC SPICE simulations with combined effects from both noisy memristor resistances and noisy crossbar wire resistances for Problem 2 (NP).

effect of both non-idealities was performed and the results are shown in Table S5. For this case each memristor's resistance was added as before Gaussian noise with standard deviations equal to 5%, 10%, and 20%; and each crossbar wire's resistance was added Gaussian noise with a standard deviation equal to 20%. For the final two sets of variability simulations, the input voltages were scaled up to ± 0.2 Volts to overcome the voltage drops caused by the wire resistances. This voltage range was chosen, because it does not disturb the conductances of the memristors Li et al. (2018).

Table S4. Average Performance (AD2S in LHS & AD2S* in RHS) results of 30 MC SPICE simulations for the implemented architectures.

		Memristance variability only (no wire resistances)			Crossbar wire resistance variability only		
	Metrics \ Variability	5%	10%	20%	5%	10%	20%
Problem 1 (NP)	MSE	1.16E-03 ± 1.45E-03	4.76E-03 ± 7.76E-03	1.43E-02 ± 2.05E-02	2.69E-04 ± 2.38E-06	2.69E-04 ± 3.63E-06	2.68E-04 ± 5.27E-06
	RSE	6.51E-02 ± 6.27E-02	1.88E-01 ± 1.92E-01	3.33E-01 ± 2.50E-01	2.04E-02 ± 1.91E-04	2.04E-02 ± 3.01E-04	2.03E-02 ± 4.36E-04
	MAE	2.60E-02 ± 1.61E-02	5.28E-02 ± 4.08E-02	9.21E-02 ± 7.34E-02	1.35E-02 ± 9.22E-05	1.35E-02 ± 1.18E-04	1.35E-02 ± 1.56E-04
	MAPE	4.80E-02 ± 2.90E-02	9.79E-02 ± 7.58E-02	1.73E-01 ± 1.38E-01	2.55E-02 ± 1.69E-04	2.55E-02 ± 2.17E-04	2.55E-02 ± 2.84E-04
	RMSE	2.97E-02 ± 1.70E-02	5.61E-02 ± 4.09E-02	9.53E-02 ± 7.32E-02	1.64E-02 ± 7.26E-05	1.64E-02 ± 1.11E-04	1.64E-02 ± 1.60E-04
	RRSE	2.31E-01 ± 1.10E-01	3.85E-01 ± 2.03E-01	5.29E-01 ± 2.34E-01	1.43E-01 ± 6.70E-04	1.43E-01 ± 1.05E-03	1.43E-01 ± 1.52E-03
	R2	9.35E-01 ± 6.27E-02	8.12E-01 ± 1.92E-01	6.67E-01 ± 2.50E-01	9.80E-01 ± 1.91E-04	9.80E-01 ± 3.01E-04	9.80E-01 ± 4.36E-04
	Metrics \ Variability	5%	10%	20%	5%	10%	20%
Problem 1 (NOAF)	MSE	1.79E-03 ± 1.86E-03	5.91E-03 ± 8.90E-03	1.78E-02 ± 1.92E-02	5.52E-04 ± 3.66E-06	5.52E-04 ± 4.96E-06	5.52E-04 ± 6.03E-06
	RSE	6.97E-02 ± 5.84E-02	1.46E-01 ± 1.53E-01	3.92E-01 ± 2.26E-01	2.92E-02 ± 1.86E-04	2.92E-02 ± 2.25E-04	2.92E-02 ± 2.44E-04
	MAE	3.34E-02 ± 1.84E-02	5.64E-02 ± 4.71E-02	1.13E-01 ± 6.48E-02	1.95E-02 ± 7.50E-05	1.95E-02 ± 8.13E-05	1.95E-02 ± 9.94E-05
	MAPE	6.04E-02 ± 3.29E-02	1.01E-01 ± 8.45E-02	2.04E-01 ± 1.18E-01	3.62E-02 ± 1.57E-04	3.62E-02 ± 1.61E-04	3.61E-02 ± 1.95E-04
	RMSE	3.81E-02 ± 1.87E-02	6.06E-02 ± 4.81E-02	1.17E-01 ± 6.46E-02	2.35E-02 ± 7.80E-05	2.35E-02 ± 1.06E-04	2.35E-02 ± 1.28E-04
	RRSE	2.45E-01 ± 1.00E-01	3.34E-01 ± 1.89E-01	5.94E-01 ± 2.02E-01	1.71E-01 ± 5.45E-04	1.71E-01 ± 6.59E-04	1.71E-01 ± 7.15E-04
	R2	9.30E-01 ± 5.84E-02	8.54E-01 ± 1.53E-01	6.08E-01 ± 2.26E-01	9.71E-01 ± 1.86E-04	9.71E-01 ± 2.25E-04	9.71E-01 ± 2.44E-04
	Metrics \ Variability	5%	10%	20%	5%	10%	20%
Problem 2 (GRU)	MSE	1.34E-03 ± 1.36E-03	3.98E-03 ± 4.92E-03	4.28E-02 ± 1.19E-01	2.07E-04 ± 3.15E-06	2.06E-04 ± 5.26E-06	2.10E-04 ± 1.05E-05
	RSE	5.41E-02 ± 5.23E-02	1.32E-01 ± 1.21E-01	3.75E-01 ± 2.69E-01	1.03E-02 ± 1.63E-04	1.03E-02 ± 2.70E-04	1.05E-02 ± 5.42E-04
	MAE	2.90E-02 ± 1.91E-02	5.19E-02 ± 3.29E-02	1.38E-01 ± 1.52E-01	1.20E-02 ± 1.14E-04	1.20E-02 ± 1.91E-04	1.21E-02 ± 3.73E-04
	MAPE	3.88E-02 ± 2.51E-02	6.95E-02 ± 4.38E-02	1.84E-01 ± 2.02E-01	1.62E-02 ± 1.34E-04	1.62E-02 ± 2.31E-04	1.64E-02 ± 4.45E-04
	RMSE	3.14E-02 ± 1.92E-02	5.41E-02 ± 3.30E-02	1.42E-01 ± 1.53E-01	1.44E-02 ± 1.10E-04	1.44E-02 ± 1.84E-04	1.45E-02 ± 3.66E-04
	RRSE	2.02E-01 ± 1.17E-01	3.27E-01 ± 1.61E-01	5.54E-01 ± 2.65E-01	1.02E-01 ± 8.00E-04	1.01E-01 ± 1.33E-03	1.02E-01 ± 2.67E-03
	R2	9.46E-01 ± 5.23E-02	8.68E-01 ± 1.21E-01	6.25E-01 ± 2.69E-01	9.90E-01 ± 1.63E-04	9.90E-01 ± 2.70E-04	9.90E-01 ± 5.42E-04
	Accuracy \ Variability	5%	10%	20%	5%	10%	20%
Problem 3 (NOG)	wafer 23	50.0%	40.0%	50.0%	100%	100%	100%
	wafer 47	100.0%	80.0%	50.0%	100%	100%	100%
	wafer 7	57.0%	43.3%	53.3%	100%	100%	100%
	wafer 3	83.3%	53.3%	46.7%	100%	100%	100%
	wafer 3838	93.3%	70.0%	56.7%	100%	100%	100%
	wafer 193	96.7%	73.3%	50.0%	100%	100%	100%
	wafer 6157	43.3%	36.7%	50.0%	100%	100%	100%
	wafer 411	100.0%	76.7%	46.7%	100%	100%	100%
	wafer 1534	93.3%	63.3%	63.3%	100%	100%	100%
	wafer 4507	100.0%	80.0%	53.3%	100%	100%	100%

Table S5. Average Performance (AD2S and AD2S*) results of 30 MC SPICE simulations with combined effects from both noisy memristor resistances and noisy crossbar wire resistances for Problems 1, 2, and 3 using NP, NOAF, GRU, and NOG architectures.

Problem 1 (NP)

Metrics \ Variability	5%	10%	20%
MSE	8.49E-4 ± 8.12E-4	4.15E-3 ± 6.47E-3	1.12E-2 ± 1.54E-2
RSE	5.83E-2 ± 4.75E-2	1.86E-1 ± 2.05E-1	3.24E-1 ± 2.36E-1
MAE	2.29E-2 ± 1.13E-2	4.83E-2 ± 3.89E-2	8.28E-2 ± 6.30E-2
MAPE	4.29E-2 ± 2.07E-2	9.03E-2 ± 7.23E-2	1.56E-1 ± 1.19E-1
RMSE	2.68E-2 ± 1.17E-2	5.19E-2 ± 3.88E-2	8.61E-2 ± 6.25E-2
RRSE	2.26E-1 ± 8.76E-2	3.77E-1 ± 2.13E-1	5.23E-1 ± 2.27E-1
R^2	9.42E-1 ± 4.75E-2	8.14E-1 ± 2.05E-1	6.76E-1 ± 2.36E-1

Problem 1 (NOAF)

Metrics \ Variability	5%	10%	20%
MSE	1.07E-3 ± 8.36E-4	3.85E-3 ± 5.14E-3	1.49E-2 ± 1.45E-2
RSE	5.39E-2 ± 3.72E-2	1.33E-1 ± 1.19E-1	3.94E-1 ± 2.45E-1
MAE	2.54E-2 ± 9.49E-3	4.67E-2 ± 3.33E-2	1.03E-1 ± 5.77E-2
MAPE	4.65E-2 ± 1.71E-2	8.46E-2 ± 6.06E-2	1.87E-1 ± 1.05E-1
RMSE	3.10E-2 ± 1.05E-2	5.23E-2 ± 3.40E-2	1.09E-1 ± 5.73E-2
RRSE	2.22E-1 ± 6.80E-2	3.31E-1 ± 1.58E-1	5.90E-1 ± 2.18E-1
R^2	9.46E-1 ± 3.72E-2	8.67E-1 ± 1.19E-1	6.06E-1 ± 2.45E-1

Problem 2 (GRU)

Metrics \ Variability	5%	10%	20%
MSE	1.36E-3 ± 1.84E-3	4.22E-3 ± 4.24E-3	3.79E-2 ± 1.05E-1
RSE	6.31E-2 ± 8.18E-2	1.63E-1 ± 1.47E-1	3.88E-1 ± 2.75E-1
MAE	2.86E-2 ± 1.95E-2	5.31E-2 ± 3.43E-2	1.32E-1 ± 1.40E-1
MAPE	3.81E-2 ± 2.55E-2	7.12E-2 ± 4.53E-2	1.77E-1 ± 1.87E-1
RMSE	3.15E-2 ± 1.94E-2	5.56E-2 ± 3.43E-2	1.36E-1 ± 1.41E-1
RRSE	2.17E-1 ± 1.29E-1	3.53E-1 ± 1.99E-1	5.73E-1 ± 2.49E-1
R^2	9.37E-1 ± 8.18E-2	8.37E-1 ± 1.47E-1	6.12E-1 ± 2.75E-1
NA	NAN	NAN	NAN
NA	NAN	NAN	NAN
NA	NAN	NAN	NAN

Problem 3 (NOG)

Accuracy \ Variability	5%	10%	20%
wafer 23	50.0%	36.7%	46.7%
wafer 47	100.0%	96.7%	73.3%
wafer 7	60.0%	36.7%	53.3%
wafer 3	100.0%	83.3%	60.0%
wafer 3838	96.7%	70.0%	53.3%
wafer 193	100.0%	96.7%	70.0%
wafer 6157	43.3%	30.0%	40.0%
wafer 411	100.0%	96.7%	66.7%
wafer 1534	93.3%	66.7%	63.3%
wafer 4507	100.0%	93.3%	66.7%

The optimized NP LSTM circuit for solving problem 1 helped to increase the overall accuracy of predictions. Compared to the work in Adam et al. (2018a), R^2 score jumped from 0.932 up to 0.995 when using more accurate circuit components – op-amps and multipliers. With these components, even the implementation with discrete weights gives high enough R^2 score of 0.975. This is a good score and promises that real memristors can give decent implementation scores.

REFERENCES

Adam, K., Smagulova, K., and James, A. P. (2018a). Memristive lstm network hardware architecture for time-series predictive modeling problems. In *2018 IEEE Asia Pacific Conference on Circuits and Systems (APCCAS)* (IEEE), 459–462

Adam, K., Smagulova, K., Krestinskaya, O., and James, A. P. (2018b). Wafer quality inspection using memristive lstm, ann, dnn and htm. In *2018 IEEE Electrical Design of Advanced Packaging and Systems Symposium (EDAPS)* (IEEE), 1–3

Brownlee, J. (2016). Time series prediction with lstm recurrent neural networks in python with keras. *Available at: machinelearningmastery.com*

Cho, K., Van Merriënboer, B., Gulcehre, C., Bahdanau, D., Bougares, F., Schwenk, H., et al. (2014). Learning phrase representations using rnn encoder-decoder for statistical machine translation. *arXiv preprint arXiv:1406.1078*

[Dataset] data, W. (2018). Wafer - time series classification website. `http://www.timeseriesclassification.com/description.php?Dataset=Wafer`. Accessed: 2018-09-30

[Dataset] Documentation, K. (2018). Recurrent layers - keras documentation. `https://keras.io/layers/recurrent/#lstm`. Accessed: 2018-09-30

Gokmen, T., Rasch, M., and Haensch, W. (2018). Training lstm networks with resistive cross-point devices. *arXiv preprint arXiv:1806.00166*

Graves, A. and Schmidhuber, J. (2005). Framewise phoneme classification with bidirectional lstm and other neural network architectures. *Neural Networks* 18, 602–610

Greff, K., Srivastava, R. K., Koutník, J., Steunebrink, B. R., and Schmidhuber, J. (2017). Lstm: A search space odyssey. *IEEE transactions on neural networks and learning systems* 28, 2222–2232

He, Q., Si, J., and Tylavsky, D. J. (2000). Prediction of top-oil temperature for transformers using neural networks. *IEEE Transactions on Power Delivery* 15, 1205–1211

Hochreiter, S. and Schmidhuber, J. (1997). Long short-term memory. *Neural computation* 9, 1735–1780

Hu, M., Strachan, J. P., Li, Z., Grafals, E. M., Davila, N., Graves, C., et al. (2016). Dot-product engine for neuromorphic computing: Programming 1t1m crossbar to accelerate matrix-vector multiplication. In *Proceedings of the 53rd annual design automation conference* (ACM), 19

Jo, S. H., Chang, T., Ebong, I., Bhadviya, B. B., Mazumder, P., and Lu, W. (2010). Nanoscale memristor device as synapse in neuromorphic systems. *Nano letters* 10, 1297–1301

[Dataset] Keras (2018). Optimizers - keras documentation. `https://keras.io/optimizers/`. Accessed: 2018-09-30

Kingma, D. P. and Ba, J. (2014). Adam: A method for stochastic optimization. *arXiv preprint arXiv:1412.6980*

Li, C., Hu, M., Li, Y., Jiang, H., Ge, N., Montgomery, E., et al. (2018). Analogue signal and image processing with large memristor crossbars. *Nature Electronics* 1, 52

Li, C., Wang, Z., Rao, M., Belkin, D., Song, W., Jiang, H., et al. (2019). Long short-term memory networks in memristor crossbar arrays. *Nature Machine Intelligence* 1, 49

Nurvitadhi, E., Sim, J., Sheffield, D., Mishra, A., Krishnan, S., and Marr, D. (2016). Accelerating recurrent neural networks in analytics servers: Comparison of fpga, cpu, gpu, and asic. In *2016 26th International Conference on Field Programmable Logic and Applications (FPL)* (IEEE), 1–4

Smagulova, K., Adam, K., Krestinskaya, O., and James, A. P. (2018a). Design of cmos-memristor circuits for lstm architecture. *arXiv preprint arXiv:1806.02366*

Smagulova, K., Krestinskaya, O., and James, A. P. (2018b). A memristor-based long short term memory circuit. *Analog Integrated Circuits and Signal Processing* 95, 467–472

Steinbuch, K. (1961). Die lernmatrix. *Kybernetik* 1, 36–45

Wang, T., Wang, C., Zhou, X., and Chen, H. (2018). A survey of fpga based deep learning accelerators: Challenges and opportunities. *arXiv preprint arXiv:1901.04988*

Wen, S., Wei, H., Yang, Y., Guo, Z., Zeng, Z., Huang, T., et al. (2019). Memristive lstm network for sentiment analysis. *IEEE Transactions on Systems, Man, and Cybernetics: Systems*

Zaremba, W., Sutskever, I., and Vinyals, O. (2014). Recurrent neural network regularization. *arXiv preprint arXiv:1409.2329*
